# Sonication is superior to scraping for retrieval of bacteria in biofilm on titanium and steel surfaces in vitro

**DOI:** 10.3109/17453670902947457

**Published:** 2009-04-01

**Authors:** Geir Bjerkan, Eivind Witsø, Kåre Bergh

**Affiliations:** ^1^Department of Orthopaedic Surgery, St. Olav's University HospitalTrondheimNorway; ^2^Department of Medical Microbiology, St. Olav's University HospitalTrondheimNorway; ^3^Department of Neuroscience, Children's and Women's Health, Norwegian University of Science and TechnologyTrondheimNorway; ^4^Department of Laboratory Medicine, Children's and Women's Health, Norwegian University of Science and TechnologyTrondheimNorway

## Abstract

**Background and purpose** Low-virulence implant infections are characterized by bacterial colonization of the implant with subsequent biofilm formation. In these cases, soft tissue biopsies often prove to be culture negative. Consequently, detachment of the causative adherent bacteria is crucial for correct microbiological diagnosis. Using an in vitro model, we compared 4 methods of biofilm sampling from metal surfaces.

**Methods** Discs of titanium and steel were incubated in the presence of *Staphylococcus aureus, Staphylococcus epidermidis, Enterococcus faecalis,* and *Propionibacterium acnes* in Mueller Hinton broth. Non-adherent bacteria were removed by repeated rinsing of the discs. 10 parallels of each disc were subjected to 1 of 4 methods for bacterial recovery: (A) sonication of the discs, (B) scraping of the discs using surgical blades followed by streaking of the blades onto agar plates, (C) scraping of the discs followed by vortex mixing of the surgical blades, and (D) scraping of the discs followed by sonication of the surgical blades. Quantitative bacterial cultures were performed for each sampling method.

**Results** With the exception of *S. epidermidis* on steel, sonication efficiently and reliably dislodged biofilm bacteria. The scraping methods employed did not detach bacteria embedded in biofilm.

**Interpretation** Scraping of metal surfaces is not an adequate method for sampling of biofilm bacteria in vitro.

## Introduction

Prosthetic joint infection (PJI) is a devastating complication occurring in about 1% and 2% of patients receiving a hip or knee prosthesis, respectively (Wilson et al. 1990, Espehaug et al. 1997, Phillips et al. 2006). Whereas diagnosis of an early postoperative PJI is usually straightforward, the diagnosis of a late or chronic PJI is notoriously difficult. Both a late, chronic PJI and aseptic prosthetic loosening present clinically with implant loosening and joint pain, and there are usually few signs of inflamation (Zimmerli et al. 2004). In late PJI, the sensitivity of culture of periprosthetic biopsies is in the range of 65–89% (Atkins et al. 1998, Spangehl et al. 1999, Pandey et al. 2000).The inadequacy of culture in this setting is probably best explained by the biofilm mode of growth of bacteria on a biomaterial surface (Gristina and Costerton 1985). Correct identification and susceptibility testing of bacteria causing a PJI is essential for the successful treatment of PJI (Hanssen and Spangehl 2004).

In order to circumvent the obstacle of the biofilm in retrieving bacteria from the implant surface, alternative strategies have been developed (Trampuz et al. 2003). In the past decade, sonication—i.e. ultrasonic energy applied directly to the biomaterial surface to disrupt adherent biofilm—has been reported to be a more reliable tool for the diagnosis of PJI (Tunney et al. 1999, Nguyen et al. 2002, Trampuz et al. 2007, Esteban et al. 2008). Sonication of a large explanted prosthesis is, however, technically demanding and carries a substantial risk of contamination during handling (Trampuz et al. 2006). Consequently, sonication has not been implemented as a standard procedure for diagnosis of chronic prosthetic joint infection.

Mechanical scraping of surfaces is often used for specimen collection and is the method of choice for certain infections, e.g. infectious keratitis (Hall and York 2004). In theory, scraping the surface of a removed implant represents a technically easy alternative for mechanical removal of adherent biofilm bacteria. It has been hypothesized that the results of scraping could be improved by either vortex mixing or sonication of the scraping product (Costerton et al. 1986). Scraping, even followed by vortex mixing or sonication of the surgical blade, is considerably less complicated than sonication of a large prosthesis. However, we are not aware of any experimental in vitro or in vivo study on scraping as a method for detachment of bacteria from metal implants. To our knowledge, only one clinical study has dealt with the use of scraping of joint prostheses in order to improve bacterial detection. Neut et al. (2003) found that as a diagnostic tool, scraping was better than conventional methods, i.e. culture of periprosthetic soft tissue biopsies. From that study, one was led to infer that bacterial detection by scraping was superior to the results presented in most sonication studies.

If equally effective, scraping would be preferable to sonication due to its technical simplicity. We here report an in vitro comparison of the recovery of biofilm bacteria from metal surfaces by sonication and by 3 different scraping techniques.

## Material and methods

**Figure 1.**
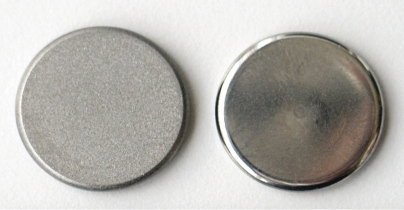
Titanium discs (left) and steel alloy discs (both from Scandinavian Customized Prosthesis AS, Trondheim, Norway) served as surfaces for biofilm formation, with roughness of Ra = 2.5 µm and Ra < 0.5 µm for titanium and steel discs, respectively. All discs were manufactured with a diameter of 17 mm and a thickness of 2 mm.

Sterilized titanium (Ti6Al4V) and steel (AIS1316-L) discs were colonized by 1 of 4 different bacterial strains (Figure 1). All strains were clinical isolates from patients with chronic PJI. The bacterial strains were identified to the species level by biotyping and/or standard microbiological procedures: *Staphylococcus aureus* (coagulase-positive, nuc-positive staphylococcus), *Staphylococcus epidermidis* (ID-32 STAPH; bioMèrièux, Marcy l'Etoile, France; profile: 166010210), *Enterococcus faecalis* (rapid ID 32 STREP; bioMèrièux; profile: 30721715171), and *Propionibacterium acnes* (rapid ID 32A; bioMèrièux; profile: 2503377604).

Confocal scanning laser microscopy (CSLM) was employed to confirm the 24-hour biofilm formation ability of each strain. 8 study groups were examined (Table 1). Bacteria were suspended in 25 mL of Mueller Hinton broth (BD, Franklin Lakes, NJ) and incubated at 35ºC until a spectrophotometric density of approximately 1 × 10^8^ colony forming units/mL (CFU/mL) had been reached in the exponential growth phase. A batch of 40 discs (one study group) was immersed in this bacterial suspension bath and incubated at 35ºC for 24 h on a gently stirring agitator (20 rpm).

**Table 1.** Overview of the study design

	Biofilm-forming bacteria							
	*S. aureus*		*S. epidermidis*		*E. faecalis*		*P. acnes*	
Experimental gruops ^a^	Titanium discs n = 40	Steel discs n = 40	Titanium discs n = 40	Steel discs n = 40	Titanium discs n = 40	Steel discs n = 40	Titanium discs n = 40	Steel discs n = 40
A	+	+	+	+	+	+	+	+
B	+	+	+	+	+	+	+	+
C	+	+	+	+	+	+	+	+
D	+	+	+	+	+	+	+	+

**^a^** Biofilm detachment was conducted as follows: (A) sonication of discs; (B) scraping of discs with direct culture by streaking the surgical blade directly onto an agar plate; (C) scraping of discs followed by vortex mixing of the surgical blade; (D) scraping of discs followed by sonication of the surgical blade.

To remove non-adherent bacteria, the discs were rinsed 6 times in sterile saline. First, the discs for each study group were placed in a sterile plastic tube (Sarstedt, Norway) containing 25 mL saline and gently vortex mixed (MS2 Minishaker; IKA Works Inc., Wilmington, NC) at 100 rpm for 10 seconds. The discs were then transferred to another tube, and the procedure was repeated twice. Each single disc was then transferred to a sterile glass test tube containing 5 mL saline and subjected to vortex mixing at 100 rpm. The single disc rinsing was also repeated 3 times.

Aliquots of 50 µL saline were incubated on agar (Merck, Darmstadt, Germany) with 5% ox blood at 35ºC for 3 days. For culture of *P. acnes*, FAA agar (Merck) was incubated in an anaerobic cabinet for 7 days. The bacteria cultured were enumerated by colony counting. The number of CFU after final rinsing was recorded as a quantitative baseline, facilitating evaluation of the different detachment methods.

Each experimental group (10 discs) was subjected to 1 of 4 methods for biofilm detachment and bacterial recovery. The experimental design is summarized in Table 1.

### Method A (sonication of discs)

Each single disc was transferred into a sterile plastic container (Minigrip, Seguin, TX) containing 5 mL saline. The container was sealed and immersed in an ultrasonic bath (TPC-120; Telsonic AG, Bronschhofen, Switzerland). Sonication at 30 kHz with a power output of 300W, as specified by the manufacturer, was performed at 37°C for 5 min. After sonication, aliquots of 50 µL were incubated as described above.

### Method B (scraping of discs with direct culture)

Thorough scraping of the complete surface of the disc was performed using a sterile surgical blade. The disc was fixed between the thumb and the index finger. 1 surgical blade was used for each disc. Seeding was done by streaking both sides of the surgical blade onto an agar plate, followed by incubation as described above.

### Method C (scraping of discs followed by vortex mixing of the surgical blade)

Scraping was performed as described for method B. After scraping, each single surgical blade was transferred into a glass test tube containing 5 mL saline before vortex mixing at 2,000 rpm for 30 seconds. After vortex mixing, an aliquot of 50 µL from each tube was incubated as described above.

### Method D (scraping of discs followed by sonication of the surgical blade)

Scraping was performed as described for method B. After scraping, each surgical blade was sonicated in 5 mL saline and aliquots of 50 µL were incubated as described above.

To prevent contamination, sterile forceps and sterile surgical gloves were used during handling of the discs and the surgical blades. All procedures were performed in a laminar airflow cabinet.

Comparison of methods A, B, C, and D was done for *S. aureus, S. epidermidis*, and *E. faecalis.* Culture of *P. acnes* requires anaerobic conditions. Due to the restrained storage capacity in an anaerobic cabinet, we were compelled to reduce the number of agar plates incubated. Hence, only methods A and C were compared for *P. acnes.*

### Statistics

The 4 methods were compared using a Kruskal-Wallis test (SPSS software). 2 group comparisons were computed with Mann-Whitney test. Statistical significance was considered at p ≤ 0.05.

## Results

**Figure 2.**
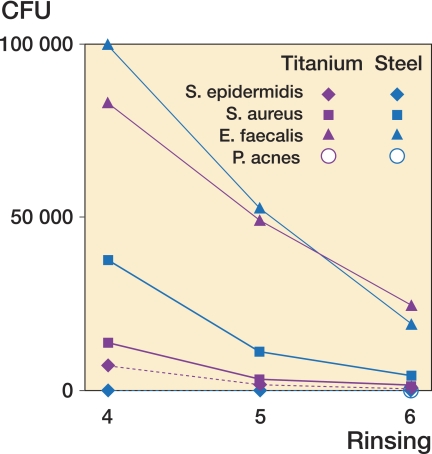
Arithmetic mean of the number of CFUs cultured after rinsing steps 4, 5, and 6 (final step) in all study groups. For 2 study groups (*P. acnes* on titanium and steel discs) culture was performed only after the final step due to limited anaerobic culture capacity.

**Figure 3.**
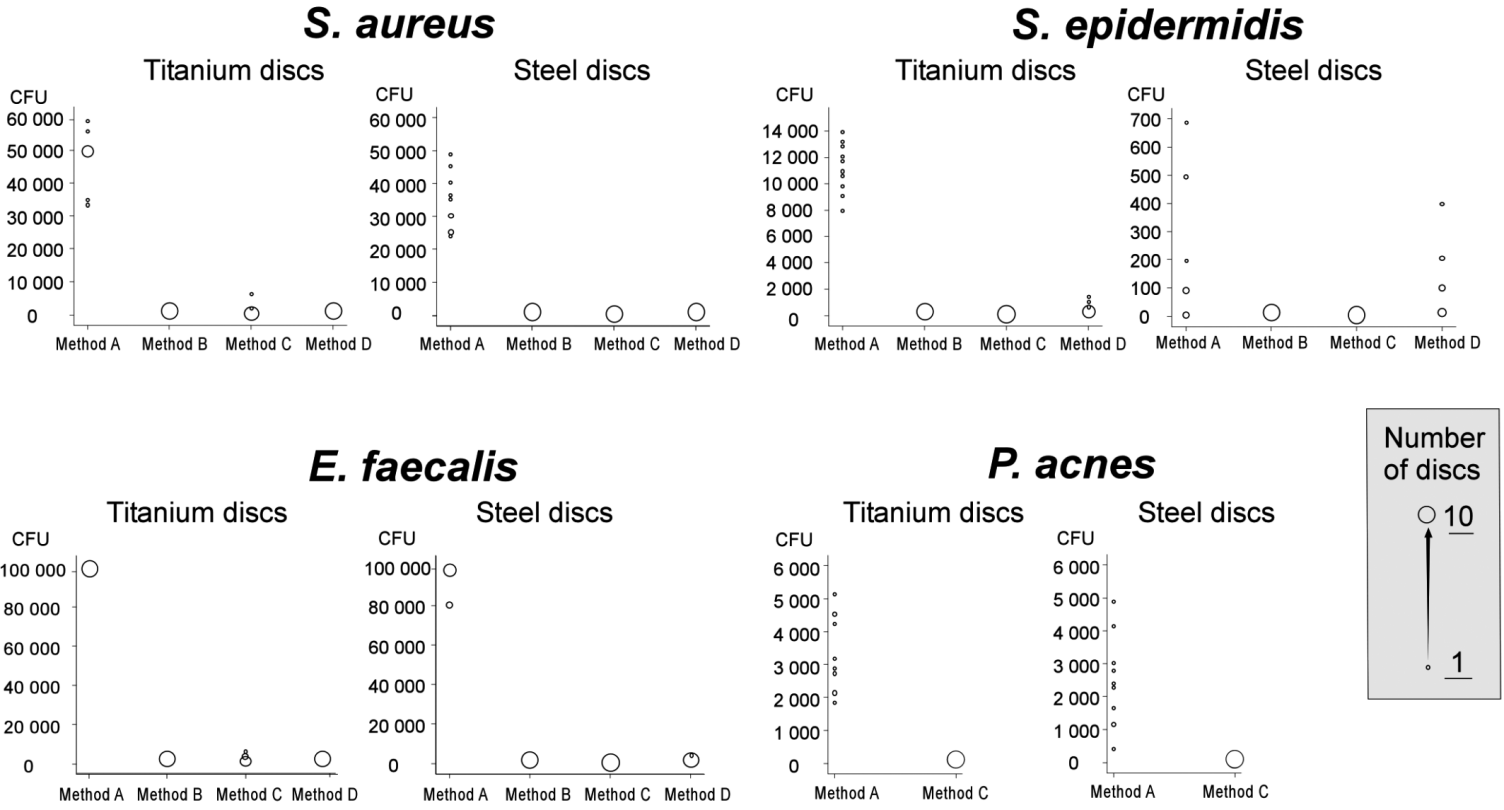
Scatter plot of number of CFUs recovered after bacterial sampling on titanium and steel discs. The bin size indicates the number of discs for which identical numbers of CFUs were retrieved.

The rinsing procedure efficiently removed non-adherent bacteria (Figure 2). The results were uniform in 7 of the 8 study groups: *S. aureus* on titanium and steel discs, *S. epidermidis* on titanium discs, *E. faecalis* on titanium and steel discs, and *P. acnes* on titanium and steel discs. Firstly, sonication of discs (method A) allowed retrieval of more bacteria than any of the scraping techniques (p < 0.001, Figure 3). Secondly, the number of CFUs detected after sonication of discs was higher compared to culture of the saline used for the final rinsing step (p < 0.001). Thirdly, all scraping techniques allowed recovery of fewer bacteria compared to the final rinsing step. Finally, only sonication recovered bacteria in 10 of 10 parallels, in contrast to scraping which yielded highly variable results (Table 2).

**Table 2.** Rate of successful bacterial recovery (10 parallels in each experimental group)

	Method			
	A	B	C	D
*S. aureus*				
Titanium	10/10	10/10	7/10	5/10
Steel	10/10	9/10	2/10	0/10
*S. epidermidis*				
Titanium	10/10	5/10	1/10	8/10
Steel	7/10	1/10	0/10	6/10
*E. faecalis*				
Titanium	10/10	10/10	10/10	4/10
Steel	10/10	9/10	4/10	6/10
*P. acnes*				
Titanium	10/10		1/10	
Steel	10/10		1/10	

For *S. epidermidis* on steel discs, bacterial recovery was generally low (Figure 3). Sonication of steel discs did not yield more *S. epidermidis* than final rinsing (p = 0.3), and bacterial growth was observed in only 7 of 10 parallels (Table 2).

There was no statistically significant difference in the number of *S. epidermidis* colonies recovered by sonication of steel discs (method A) and scraping followed by sonication of the surgical blade (method D) (p = 0.4; Figure 3). However, both method A and method D allowed recovery of more bacteria than scraping and direct culture (method B) and scraping followed by vortex mixing of the surgical blade (method C) (p < 0.05).

## Discussion

Our study was undertaken to retrieve bacteria from biofilms on titanium and steel surfaces using an in vitro model, and to compare different methods of biofilm disruption for subsequent culture. With the exception of *S. epidermidis* on steel, our findings clearly show that sonication is capable of detaching bacteria in this model. The various scraping methods proved to be insufficient by demonstrating a lower yield and highly in consistent results. This conclusion is based on the concept that in order to disrupt bacteria from a biofilm, the number of bacteria recovered must be increased compared to what can be observed after the final step of repeated washing.

These results also present circumstantial evidence of the successful establishment of biofilms on the metal surface. The one study group that was different quantitatively was *S. epidermidis* on steel. Here, we cannot rule out the possibility that there was a poorly developed biofilm containing a low number of bacteria—even though in a separate study biofilm matrix was evident by CSLM after calcofluor white staining. An alternative interpretation is that not even sonication effectively dislodges an *S. epidermidis* biofilm from steel surfaces.

There are several methodological considerations relevant to our study. A number of in vitro models for biofilm formation exist (Mombelli 1999). We used a dynamic version of a closed system biofilm method with standard conditions of fluid motion and temperature to culture biofilms simultaneously on 40 discs (Christensen et al. 2000, Sissons et al. 2000). The time allowed for biofilm formation also varies considerably. Overnight (18-hour), 24- or 48-hour incubation periods have been recommended for biofilm formation in vitro (An and Friedman 1997). Most investigators use incubation periods from just a few hours up to 24 hours (Christensen et al. 1987, Tollefson et al. 1987, Olson et al. 1988, Khardori and Yassien 1995, Vandecasteele et al. 2003, van der Borden et al. 2004). The bacterial species we studied, *S. aureus, S. epidermidis, E. faecalis,* and *P. acnes*, are all common causative microbes in chronic prosthetic infections (Tsukayama et al. 1996, Berbari et al. 1998, Lentino 2003, Zeller et al. 2007). A disadvantage of using clinical isolates as opposed to reference strains is the incomplete knowledge of their detailed biofilm characteristics and limited application in laboratories (Oliveira et al. 2007). The clinical strains we employed had a priori demonstrated their ability to establish clinically relevant biofilm infections on joint prostheses.

In routine orthopedic surgery, several different foreign materials are regularly implanted, e.g. bone cement, polyethylene compounds, and different metal alloys. Biomaterials have different affinities for bacteria (Oga et al. 1993). In general, an increase in surface roughness enhances bacterial colonization and early biofilm formation (Arnold and Bailey 2000). Both the surface roughness of the discs and the metal alloy we used to make them were similar to those found in commonly used hip prostheses (Furnes et al. 2007).

The purpose of sonication to improve the diagnosis of PJI is to detach biofilm bacteria on the implant surface using ultrasonic energy (above 20 kHz). For subsequent culture, the bacteria must still be viable after sonication. Before the study, we had sonicated each of the 4 bacterial strains for 20 min without observing any bactericidal effect (data not shown). Kobayashi et al. (2007) recommended a sonication time of between 1 and 5 min as being ideal for dislodging biofilm bacteria without affecting bacterial viability. Investigators using sonication should report the manufacturer and model number of the equipment, the output power, oscillation frequency, reaction volume, fluid temperature, and sonication time (Christensen et al. 1995). Sonication at about 50 Hz is sometimes reported (Tunney et al. 1999, Nguyen et al. 2002, Bayston et al. 2007). Reporting this pretransduced electric frequency is in our opinion misleading and confusing, because no dislodgement of biofilm will occur at 50 Hz.

In a clinical context, sonication of a large explanted prosthesis is technically demanding and carries a significant risk of contamination during handling. Thus, the encouraging results obtained from scraping the prosthesis and directly inoculating the agar plate indicated that scraping is a beneficial method due to its technical simplicity (Neut et al. 2003). However, these results have not been reproduced by other research groups. The results from our in vitro study indicate that scraping, even followed by post-scraping procedures for disrupting the biofilm, is not an efficient technique for dislodgement of biofilm bacteria.

To conclude, with the exception of *S. epidermidis* on steel, we have demonstrated that sonication efficiently dislodges bacteria from biofilms generated in vitro on titanium and steel surfaces. Scraping of metal surfaces cannot be recommended as a method for diagnosis of biofilm-related infection. Further studies on sonication are warranted, as are studies comparing methods for biofilm sampling in vivo.
